# The heterogeneous effects of the great recession on informal care to the elderly

**DOI:** 10.1007/s10754-022-09325-w

**Published:** 2022-03-07

**Authors:** Jesús M. Carro, Elizaveta Pronkina

**Affiliations:** 1 Department of Economics, Universidad Carlos III de Madrid, Getafe, Spain; 2 LEDa-LEGOS, Université Paris-Dauphine - PSL, Paris, France

**Keywords:** Informal care, Great recession, Unobserved heterogeneity, I18, J14, C2

## Abstract

This paper studies the role of unobserved factors to measure the impact of the economic downturn on informal care availability to the elderly in Europe. We use the Survey of Health, Ageing and Retirement in Europe (SHARE), which allows controlling for socio-demographic variables. Our results show that the impact of the Great Recession on care receipt depends not only on observed, but also on unobserved characteristics. For 21% of the sample, the effect is three to four times larger than the average effect for the entire sample. For 57% of the sample, there is no effect of the economic crisis, and this is related to unobservable factors. In our estimation process, we are able to characterize how this unobserved heterogeneity correlates with the observable variables. Moreover, we show that if the unobserved heterogeneity in the effect of the crisis is ignored, then we are not able to capture that there is no effect for more than half of the individuals, even if we allow for unobserved heterogeneity in the intercept of the model and for the heterogeneous effect of the crisis based on observables.

## Introduction

Long-term care expenditures do not always cover all care loads, and elders often rely on informal care later in their lives. Thus the provision of informal care remains essential for a significant part of the population. However, the availability of informal care depends not only on the needs of the elderly but also on the possibility and availability of caregivers. This availability can change with economic conditions. How does informal care respond to the economic crisis? Recent scholars answer this question by exploiting the macroeconomic downturn caused by the Great Recession. (Costa-Font et al., 2016) find that the supply of informal care is countercyclical in Europe. In the US, (Mommaerts & Truskinovsky, 2020) show that informal care provided by spouses is procyclical, while from children is countercyclical.

In this paper, we study the impact of a macroeconomic downturn on informal care availability in the presence of unobserved heterogeneous effects. In this context, some variables like family ties, underlying health conditions of carereceiver or the profile of potential caregivers are often unavailable or unobserved but likely impact the level of informal care availability and the intensity of how the Great Recession changed the probability of receiving informal care. Accordingly, in the paper, we directly account for such unobserved factors. Both recent articles acknowledge the importance of heterogeneity based on observables. Costa-Font et al. (2016) show that the impact of the Great Recession on care available to the elderly is twice larger for individuals without children, and the findings vary across long-term care (LTC) systems of European countries. Further, (Mommaerts & Truskinovsky, 2020) uncover the heterogeneity based on educational attainment and age of care receivers. Accordingly, with this study, we add to the existing literature by showing that unobserved characteristics matter when characterizing the impact of the economic crisis on receiving informal care.

Our identification strategy considers a non-linear model of our binary outcome variable and allows for the heterogeneous impact in both the availability of care and the impact of the Great Recession in Europe. We use finite discrete mixtures to model the unobserved heterogeneity. Using the Survey of Health, Ageing and Retirement in Europe (SHARE), we find evidence for observed and unobserved heterogeneity. Looking at the impact of the crisis on care receipt, we find two unobserved types of individuals. The impact of the crisis is large and countercyclical among 21% of individuals. At the same time, it is not significant for 57% of individuals that would be misrepresented by a conclusion about the countercyclicality of informal care based only on the average effect over the entire sample. An individual in the most affected unobserved group is more likely to be older, with lower education, and live without a partner. Disregarding heterogeneity based on unobservables masks a sizeable countercyclical effect for this 21% of the sample and gives a small impact of the crisis. Our results highlight the importance of unobservable factors in measuring the impact of the economic downturn on informal care.

## Data

This paper exploits the SHARE dataset.^1^ It provides socio-demographic and economic information about individuals aged above 50. The sample includes countries that participate in the survey from 2004 to 2013: wave 1 (2004/05); wave 2 (2006/07); wave 4 (2010/11); and wave 5 (2013). In total, there are eleven countries included in the analysis: Austria, Belgium, the Czech Republic, Denmark, France, Germany, Italy, the Netherlands, Poland, Spain, and Sweden.

We restrict our sample to individuals who are present at least in two waves. In total, there are 101646 individual - wave observations.^2^ Table 1 shows the descriptive statistics of variables used in this study.

The main outcome variable captures informal care receipt inside or outside the household from any family member, friend or neighbor during the last 12 months. Since the question about care outside the household is asked at the family level, and it is not clear who is the member of the couple receiving care, we impute care receipt to the individual level exploiting information about respondent’s and spousal activity limitations, mobility limitations, and living in a nursing home status. As far as it is possible, our imputation procedures follow the same criteria as Costa-Font et al. (2016), based on the details given in their paper. When there were doubts about to whom to assign care, samples using several alternatives were constructed. Since it affected only a very small proportion of our sample, the results did not change, and we report results using only one of those criteria. The specific details of the SHARE questions used and of our imputation procedures are provided in the Appendix.

**Table 1:** Descriptive statistics

Variable	Mean	SD	Observations
Outcome			
Receipt of informal care	0.20	0.40	101646
Controls			
Age	66.13	9.84	101646
Elderly (age$\ge$70)	0.32	0.47	101646
Female	0.56	0.50	101646
Living with a partner	0.72	0.45	101646
Tertiary education	0.23	0.42	101646
Secondary education	0.48	0.50	101646
Having a child	0.91	0.29	101646

Further, we consider the measure of the crisis based on the country-specific change in the unemployment rate during the GDP drop. In waves 1 and 2 (before the Great Recession), crisis equals 0, whereas, in the rest of the waves, it is equal to the value from Table III in Costa-Font et al. (2016). The original variables are computed based on statistics on Eurostat.

## Model and methods

To estimate the impact of the depth of the economic crisis on receiving informal care, we use the following model:

1 $$\begin{aligned} y_{it}&= \mathbbm {1}(\alpha _{i0}+ \alpha _{i1} crisis_{k(i)t} + \beta _1 crisis_{k(i)t} elderly_{it} + \beta _2 age_{it} + \beta _3 age^2 _{it} + \beta _4 partner\_in_{it} \nonumber \\&\quad + \beta _5 tertiary_{it} + \beta _6 secondary_{it} + \beta _7 child_{it} + \beta _8 female_i + \mu _{k(i)} + \nu _t+\epsilon _{it}\ge 0), \end{aligned}$$

where $y_{it}$ is a binary indicator of informal care receipt by individual *i* at wave *t*; *k*(*i*) denotes the country *k* of individual *i*; $crisis_{k(i)t}$ measures the severity of the Great Recession in each country *k* at period *t*. The variable $elderly_{it}$ is a binary indicator of being above 70 years old, $partner\_in_{it}$ is an indicator to live with a partner, $tertiary_{it}$ and $secondary_{it}$ are education indicators, leaving ‘less than secondary education’ as the reference level, $child_{it}$ is a binary indicator for having a child, and $female_{i}$ is a female indicator. The country fixed effects are $\mu _{k(i)}$, and $\nu _{t}$ are wave fixed effects. $\epsilon _{it}$ is an iid error that follows a standard normal distribution. With respect to the model in Costa-Font et al. (2016), in addition to taking into account the non-linear nature of the dependent variable,^3^ we allow for heterogeneous effects of the crisis across individuals, both in observable and unobservable characteristics.

The effect of the crisis is allowed to be different across individuals depending on unobservable characteristics, $\alpha _{i1}$, and on being elderly, $\beta _{1}$, which is an observable characteristic. The observed heterogeneity in the cyclicality of informal care based on care-receiver’s age has been already documented by Mommaerts and Truskinovsky (2020) using US data. Up to our knowledge, unobserved heterogeneity on the effect of the crisis has not been considered yet, and it could be important if there are large differences in the effect of the crisis across individuals. There are several structural (economic) sources for why there can be unobserved heterogeneity in the response of informal care to the Great Recession. One source is different family ties. These can affect not only the level of informal care, but also the intensity of how the Great Recession changes the probability of receiving informal care. Individuals with different family ties may be affected differently by the crisis. See, for example, (Bolin et al. 2008) for arguments on the importance of family ties for informal care. Related to this, the heterogeneous response to the crisis can come from heterogeneous effects of the crises over caregivers’ labor market status and caregiving choices. Mommaerts and Truskinovsky (2020) finds, in its analysis of caregivers’ decision problem, that the business cycle has a different effect on informal caregiving decisions depending on age, relation to the care-receiver, education, marital status, and gender of the caregiver. Since in SHARE we do not have information on these variables for all the potential caregivers providing informal care to the care-receivers of our sample, this heterogeneity on the effect of the crisis is unobserved. Another unobserved aspect that can lead to different effects of the crisis is the underlying health conditions, like the propensity to suffer from some diseases. Health may worsen more with the crisis—see Bucher-Koenen and Mazzonna (2013) and Costa-Font et al. (2016)—for those with a higher predisposition, and this may lead to a larger effect of the crisis on the probability of receiving informal care for those individuals.

Therefore, and different from the canonical panel data model with individual fixed effects where only the intercept $\alpha _{i0}$ is allowed to be heterogeneous across individuals, we also allow the effect of the crisis $\alpha _{i1}$ to be heterogeneous in an unobservable way. This goes in line with the importance, for correct estimation and inference on the marginal effects of variables, of allowing for more unobserved heterogeneity than only in the intercept showed in Browning and Carro (2010) for discrete choice models. We model unobserved heterogeneity using discrete finite mixtures. This assumes that the permanent unobserved factors follow a distribution that can take only a given finite number of values (often called types). Those values, and the probability of taking them (or proportion of each type), are estimated jointly with the rest of the parameters of the model. The identification of this distribution, as any other distribution of the unobserved heterogeneity, is only possible with a panel. This way of controlling for unobserved heterogeneity, though it restricts the number of possible values to be discrete and finite, allows for very flexible form of the distribution of the heterogeneity. It was popularized in econometrics by Heckman and Singer (1984), that shows how it can approximate a continuous distribution in a duration model context. Since then it has been used in several discrete choice models, especially in structural estimation. Deb and Trivedi (1997) and Halliday (2008) are two examples of the use of finite discrete mixtures in other health economics models, with the latter allowing for the slope, as well as the intercept, to be heterogeneous, as we do in our model.

Formally, we assume that respondents are of one of two possible types, so that $\left( \alpha _{i0},\alpha _{i1}\right) =\left\{ \left( \alpha _{10},\alpha _{11}\right) ,\left( \alpha _{20},\alpha _{21}\right) \right\}$.^4^ This allows capturing unobserved heterogeneity in the availability of care and in the impact of the Great Recession on care receivers, and for free correlation between them. The probability of each type is defined as follows,

2 $$\begin{aligned}&\Pr \left( \left. \text {type}=1\right| X_{i}\right) \end{aligned}$$

3 $$\begin{aligned}&\quad =\frac{\exp \left( \begin{array} [c]{c} \gamma _{10}+\gamma _{11}\overline{crisis_{k(i)}}+\gamma _{12}\overline{age_{i} }+\gamma _{13}\overline{partner\_in_{i}}\\ +\gamma _{14}\overline{tertiary_{i}}+\gamma _{15}\overline{secondary_{i}} +\gamma _{16}\overline{child_{i}}+\gamma _{17}female_{i} \end{array} \right) }{1+\exp \left( \begin{array} [c]{c} \gamma _{10}+\gamma _{11}\overline{crisis_{k(i)}}+\gamma _{12}\overline{age_{i} }+\gamma _{13}\overline{partner\_in_{i}}\\ +\gamma _{14}\overline{tertiary_{i}}+\gamma _{15}\overline{secondary_{i}} +\gamma _{16}\overline{child_{i}}+\gamma _{17}female_{i} \end{array} \right) } \nonumber \\&\Pr \left( \left. \text {type}=2\right| X_{i}\right) =1-\Pr \left( \left. \text {type}=1\right| X_{i}\right) , \end{aligned}$$

where the upper bar represents the within groups average, that is the average over the four waves for each individual.

We use maximum likelihood (ML) to estimate the parameters of the probability to receive care, Eq. [1], and the distribution of types, Eqs. [2] and [3]. The probabilities that form the likelihood are given by

4 $$\begin{aligned} \Pr \left( y_{it}=1|X_{it}\right)&=\Pr \left( y_{it}=1|X_{it},type=1\right) *\Pr \left( type=1|X_{it}\right) \nonumber \\&+\Pr \left( y_{it}=1|X_{it},type=2\right) *\Pr \left( type=2|X_{it}\right) , \end{aligned}$$

where, from Eq. [1],

5 $$\begin{aligned} \Pr ( y_{it}&=1|X_{it},type=j) \nonumber \\&\quad =\varPhi \left( \begin{array} [c]{c} \alpha _{j0}+\alpha _{j1}crisis_{k(i)t}+\beta _{1}crisis_{k(i)t}elderly_{it}+\beta _{2}age_{it}+\beta _{3}age_{it}^{2}\\ +\beta _{4}partner\_in_{it}+\beta _{5}tertiary_{it}+\beta _{6} secondary_{it}\\ +\beta _{7}child_{it}+ \beta _8 female_i+\mu _{k(i)}+\nu _{t} \end{array} \right) \end{aligned}$$

and $\Pr \left( type=j|X_{it}\right)$, where $j=1,2$, is in Eqs. [2] and [3]. $\varPhi (\cdot )$ is the cumulative distribution function of the standard normal distribution. Then, the log-likelihood is

$$\begin{aligned} \begin{aligned} l\left( \pmb {\alpha },\pmb {\beta ,\gamma }\right) =\sum _{i=1}^{N} \sum _{t=1}^{T}\ln \left( \Pr \left( y_{it}=1|X_{it}\right) \right) . \end{aligned} \end{aligned}$$

The variance-covariance matrix of the ML Estimation is obtained as minus inverse of the Hessian of $l\left( \pmb {\alpha },\pmb {\beta ,\gamma }\right)$.

## Results

Table 2 reports the coefficients for the probability of care receipt and of being type 1. Even though we cannot interpret the magnitude of the coefficients, we can comment on the sign of those factors (*Panel I* in Table 2). From there, the impact of the crisis is larger for older individuals, which is in line with Mommaerts and Truskinovsky (2020), who find observed heterogeneity in the age of care receiver. $\left( \alpha _{10},\alpha _{11}\right)$ are statistically different from $\left( \alpha _{20},\alpha _{21}\right)$, which means that there is unobserved heterogeneity in the effect of the crisis. Care receipt and age follow a U-shaped relation, with the elders being more likely to receive care in our sample. Living with a partner in the same household decreases the probability of receiving care, reflecting that those living with a partner tend to have better health.^5^ Compared to individuals with primary education, having secondary education decreases the probability of receiving care. Since children are one of the primary caregivers, it is not surprising that we find an increase in care receipt if an individual has a child.

**Table 2:** Maximum likelihood estimates of parameters

Parameter	Variable	Estimate	Standard error
*Panel I*: Receipt of informal care			
Unobserved type effects			
*Type 1:*			
$$\alpha _{10}$$	Constant	$$5.0528^{***}$$	0.2557
$$\alpha _{11}$$	Crisis	$$0.0605^{***}$$	0.0122
*Type 2:*			
$$\alpha _{20}$$	Constant	$$3.7588^{***}$$	0.2547
$\alpha _{21}$	Crisis	0.0032	0.0064
Observed characteristics			
$$\beta _{1}$$	Crisis $$*$$ Elderly	$$0.0320^{***}$$	0.0058
$$\beta _{2}$$	Age	$$-0.1497^{***}$$	0.0074
$$\beta _{3}$$	Age squared	$$0.0012^{***}$$	0.0001
$$\beta _{4}$$	Living with a partner	$$-0.4124^{***}$$	0.0247
$\beta _{5}$	Tertiary education	0.0217	0.0385
$$\beta _{6}$$	Secondary education	$$-0.1100^{***}$$	0.0371
$\beta _{7}$	Having a child	$0.0478^{*}$	0.0251
$$\beta _{8}$$	Female	$$0.1370^{***}$$	0.0291
Country and wave dummies are included.			
*Panel II*: Probability of type 1, parameters			
$$\gamma _{10}$$	Constant	$$-3.2267^{***}$$	0.4755
$$\gamma _{11}$$	$$\overline{\text {Crisis}}$$	$$-0.1212^{***}$$	0.0256
$$\gamma _{12}$$	$$\overline{\text {Age}}$$	$$0.0369^{***}$$	0.0055
$$\gamma _{13}$$	$$\overline{\text {Living with a partner}}$$	$$-0.3623^{***}$$	0.0895
$$\gamma _{14}$$	$$\overline{\text {Tertiary education}}$$	$$-0.5978^{***}$$	0.1392
$\gamma _{15}$	$\overline{\text {Secondary education}}$	$-0.1918^{*}$	0.11148
$\gamma _{16}$	$\overline{\text {Having a child}}$	$-0.1340$	0.1507
$\gamma _{17}$	Female	0.0817	0.1001
Log-likelihood		$-45833.53$	
No. of units		38601	
No. of observations		101646	

*Note*: In *Panel II*, the upper bar corresponds with the within groups average for each individual. Asterisks indicate the estimate is significantly different from zero at *10%; **5%; ***1%. Standard errors are estimated using the inverse of Hessian matrix of the log-likelihood

*Panel II* in Table 2 shows that observables are significantly associated with the probability of being of each type. Based on it, we can discuss the profile of individuals. The probability of being type 1 is higher for elders, with primary education, and who do not live with a partner. Interestingly, having a child does not statistically aggravate inequalities in the receipt of informal care as the probability of being type 1 does not change.

To interpret the magnitude of the effect of the Great Recession, we compute the average increase in the probability of receiving informal care as a consequence of an increase in our measure of the economic crisis, for different groups of individuals. These are generally named as the average marginal effects (AME). We also compute and the average probabilities of receiving care for each type. These are reported in Table 3. For that, we fix the values of the covariates in the last period before the crisis, wave 2. For example, our estimate of the AME for all individuals being type 1, the first number reported in Table 3 is given by

$$\begin{aligned}&\widehat{AME_{type1}}=\frac{1}{N}\sum _{i=1}^{N}\frac{\partial \widehat{\Pr }\left( y_{i2}=1|X_{i2},type=1\right) }{\partial crisis_{k(i)2}}=\frac{1}{N} \sum _{i=1}^{N}\Bigl [\left( \widehat{\alpha }_{11}+\widehat{\beta }_{1} elderly_{i2}\right) \\&*\phi \left( \begin{array} [c]{c} \widehat{\alpha }_{10}+\widehat{\alpha }_{11}crisis_{k(i)2}+\widehat{\beta } _{1}crisis_{k(i)2}elderly_{i2}+\widehat{\beta }_{2}age_{i2}+\widehat{\beta } _{3}age_{i2}^{2}\\ +\widehat{\beta }_{4}partner\_in_{i2}+\widehat{\beta }_{5}tertiary_{i2} +\widehat{\beta }_{6}secondary_{i2}\\ +\widehat{\beta }_{7}child_{i2}+\widehat{\beta }_{8}female_{i}+\widehat{\mu }_{k(i)}+\widehat{\nu }_{2} \end{array} \right) \Bigl ] \end{aligned}$$

where $\phi \left( \cdot \right)$ is the standard normal density function. The standard errors of the AMEs are obtained using the delta method from the Variance-Covariance matrix of the maximum likelihood estimator of the model’s parameters.

**Table 3:** Average marginal effect of the crisis on care availability in wave 2 for each unobserved type

	Heterogeneity in $\alpha _{i0}$ and $\alpha _{i1}$	
	Type 1	Type 2
*Panel I*: All individuals		
AME	$$0.0263^{***}$$	$$0.0029^{*}$$
	(0.0048)	(0.0016)
$\overline{\Pr \left( y=1\right) }$	0.5267	0.1219
$\overline{\Pr \left( \text {type}=j\right) },$ $j=1,2$	0.2069	0.7931
*Panel II*: Individuals younger than 70 (68.73% of the sample)		
AME	$$0.0233^{***}$$	0.0005
	(0.0047)	(0.0011)
$\overline{\Pr \left( y=1|age<70\right) }$	0.4838	0.0935
$\overline{\Pr \left( \text {type}=j|age<70\right) }$	0.1641	0.8359
*Panel III*: Individuals older than 70 (31.27% of the sample)		
AME	$$0.0331^{***}$$	$$0.0083^{***}$$
	(0.0054)	(0.0025)
$\overline{\Pr \left( y=1|age\ge 70\right) }$	0.6211	0.1814
$\overline{\Pr \left( \text {type}=j|age\ge 70\right) }$	0.3011	0.6989

*Note* $\overline{\Pr \left( y=1\right) }$ is the average predicted of probability of care receipt. Standard errors are in parenthesis. Asterisks indicate the estimate is significantly different from zero at *10%; **5%; ***1%

After controlling for observables, we find that informal care receipt is countercyclical for individuals of type 1. For type 1, the probability of informal care increases on average in 2.63 percentage points in the entire population of type 1, and 3.31 percentage points for those older than 70, as reported in Table 3. This group, older than 70 and type 1, has the biggest effect of economic downturn on their probability of receiving care and represents 9.4% of the individuals.^6^ The group that is type 1 and younger than 70 represents 11% of the population and has an average effect of 0.0233. All these estimated effects are statistically significant at a 1% level. By contrast, care receipt is almost not affected by the economic downturn for individuals of type 2, whose estimated AME is small in magnitude, 0.0029. The AME of the crisis for type 2 is small even for those older than 70. For those younger than 70 and type 2, the estimated average effect, 0.0005, is not statistically different from zero. This group (type 2 and younger than 70) with a zero effect represents 57% of the sample.^7^

The AME for the entire population over the two types is equal to 0.0077, and it is statistically significant. However, in this case where more than half of the population is not affected by the crisis and for 21% the effect is more than three times larger than this average effect, the AME for the entire population is not a very informative measure. This is one of those situations in which, if our interest is on studying the cyclically of informal care, reporting only the average effect will mask large differences across population, including no cyclical effect for most of the sample.

Looking at average probabilities of receiving care, individuals of type 1 are more likely to receive care than individuals of type 2; the average probability is equal to 0.53 and 0.12, respectively (Table 3 ). For those older than 70, those average probabilities are higher, 0.62 and 0.18 respectively, in accordance with the positive effect of age on the probability of receiving care.

## Further discussion about heterogeneity

The average (prior) probability of being of type 1–calculated using Eq. 2—is, on average, 0.21. Calculating this probability for each individual, taking into account their value of the covariates that enter into Eq. [2], this probability is hardly above 0.50 for any individual in the sample. However, we can consider the observed outcome history, $Y_{i}$, and compute the posterior probability of being of type 1, to be able to identify better the type of each individual. Using the Bayes rule, the posterior probability of type 1 is:

$$\begin{aligned} \Pr \left( \left. \text {type}=1\right| X_{i},Y_{i}\right) =\frac{\Pr \left( \left. Y_{i}\right| X_{i},\text {type}=1\right) \Pr \left( \left. \text {type}=1\right| X_{i}\right) }{\Pr \left( \left. Y_{i}\right| X_{i}\right) } \end{aligned}$$

where the first term of the numerator is in [5], the second term of the numerator is the "prior" probability in [2], and the denominator is in [4]. Looking at the posterior probabilities, there are 13% of individuals whose probability of being of type 1 is higher than 0.50. In the sample, those individuals most likely to be of type 1, compared with the rest of the population, are older (72 versus 65 years old on average), less often have a partner in a household (54% versus 75%), and have less education: only 13% (43%) of them have tertiary (secondary) education, whereas among those of type 2 those proportions are 24% and 50%. Finally, individuals of type 1 are more frequently women (61% versus 55%) and slightly less likely to report having a child than type 2 (87% versus 91%). This conclusion is in line with the interpretation of the probability to be of each type and observable from *Panel II* in Table 2.

Another interesting aspect of the estimated parameters is that there is positive correlation between $\alpha _{i0}$ and $\alpha _{i1}$. Given that $\left( \alpha _{10},\alpha _{11}\right)$ are greater than $\left( \alpha _{20},\alpha _{21}\right)$, care receipt is more prevalent among type 1 individuals and, at the same time, the impact of crisis is larger for them. This means that the Great Recession magnified inequalities in the receipt of informal care due to either increase in availability or need thereof. To the extent unobserved heterogeneity can be capturing underlying health conditions and propensity to suffer some medical conditions and limitations, this would mean higher propensity implies receiving more informal care ceteris paribus (larger $\alpha _{i0}$) and at the same time are more affected by the crisis (larger $\alpha _{i1}$) because the crisis worsens relatively more the health conditions of those with a higher predisposition to suffer medical conditions. Likewise, those whose family ties make them to receive more informal care ceteris paribus, are also more affected by the crisis. Additionally, the effect being smaller for type 2 and the fact that individuals of type 2 are more likely to have a partner in the household, as we have found, is in line with the unobserved heterogeneity being capturing a heterogeneous effect of crisis on caregivers: the care provided by spouses is procyclical, according to Mommaerts and Truskinovsky (2020); and this procyclical effect would compensate the other ways in which the probability of receiving informal care can increase during economic downturns.

When we allow for three unobserved types in the analysis, the likelihood increases, but parameters $\left( \alpha _{j0},\alpha _{j1}\right)$ of types 2 and 3 are not statistically different at a 10% level of significance, i.e. we cannot reject the null hypothesis that $\alpha _{20}=\alpha _{30}$ and $\alpha _{21}=\alpha _{31}$ at a 10% level. Additionally, the BIC criteria, that penalizes the number of parameters, prefers the specification with 2 unobserved types over a specification with 3 types. We also allowed for a heterogeneous effect of the crisis based on the education level of the individual, as Mommaerts and Truskinovsky (2020) did, but this differential effect was not statistically significant in our sample, in the model with two types, and this interaction with education was discarded.

Next, we repeat the analysis ignoring unobserved heterogeneity in the effect of the crisis, but allowing two unobserved types in the intercept, $\alpha _{i0}$, and keeping the heterogeneous effect based on the age. The estimated average marginal effect for the entire population is 0.0060. This overall AME is close (within the confidence interval) to the AME estimated in Costa-Font et al. (2016) using a linear specification with heterogeneity in $\alpha _{i0}$, and to our estimate with heterogeneity in both $\alpha _{i0}$ and $\alpha _{i1}$, 0.0077. However, as Table 4 shows, it fails to capture that there is a group representing more than half of the population (type 2 younger than 70) for which the effect is not statistically different from zero. The same happens if we use a continuous normal distribution for $\alpha _{i0}$ but imposing that $\alpha _{i1}$ is homogeneous. Furthermore, even though the observable heterogeneity for different age groups in the effect of crisis is maintained, omitting unobserved heterogeneity in $\alpha _{i1}$ cannot capture the magnitude of crisis for those of type 1 older than 70 years. The effect for this group in Table 4 is half of the effect in Table 3, 0.0162 vs. 0.0331.

**Table 4:** Average marginal effect ignoring unobserved heterogeneity in the impact of the crisis

	Heterogeneity only in $\alpha _{i0}$		No unobserved
	Type 1	Type 2	heterogeneity
AME in wave 2	$$0.0090^{***}$$	$$0.0051^{***}$$	$$0.0052^{***}$$
	(0.0026)	(0.0014)	(0.0012)
AME in wave 2 for	$$0.0057^{**}$$	$$0.0026^{**}$$	$$0.0026^{**}$$
those younger than 70	(0.0023)	(0.0011)	(0.0012)
AME in wave 2 for	$$0.0162^{***}$$	$$0.0106^{***}$$	$$0.0109^{***}$$
those older than 70	(0.0036)	(0.0024)	(0.0015)
$\overline{\Pr \left( y=1\right) }$	0.5436	0.1240	0.2038
$\overline{\Pr \left( \text {type}=j\right) },$ $j=1,2$	0.1958	0.8042	
$\overline{\Pr \left( \text {type}=j|age<70\right) }$	0.1503	0.8497	
$\overline{\Pr \left( \text {type}=j|age\ge 70\right) }$	0.2957	0.7043	

*Note*: $\overline{\Pr \left( y=1\right) }$ is the average predicted probability of care receipt. Asterisks indicate the estimate is significantly different from zero at *10%; **5%; ***1%

Finally, ignoring unobserved heterogeneity all together (but keeping observed heterogeneity with age), reported in the last column in Table 4, not only fails to capture that more than half of the individuals are not affected by the economic crisis, but also underestimates the AME for the entire sample. This specification estimates and AME of 0.0054, whose 95% interval, (0.0032, 0.0075), does not include the AME estimated with our model, 0.0077.

## Conclusion

Recent articles have studied the cyclicality of informal care receipt but have not explored the role of unobserved factors. We add to the existing literature by estimating the impact of unobserved factors in the effect of economic crisis and discussing the profile of the more affected individuals in Europe. There is unobserved and observed heterogeneity in the data.

Based on the unobserved heterogeneity, the Great Recession, measured by the change in the employment rate, did not have any effect for 57% of the sample, which corresponds to most of those younger than 70 years old. Furthermore, among the 43% individuals with a positive and significant effect of the crisis, there are two groups, representing 9.4% and 11% of the total sample, whose AME is four and three, respectively, times larger than the average marginal effect for the entire sample. Ignoring the existence of the unobserved heterogeneity fails to recognize these effects of the Great Recession. Accordingly, future research considering the impact of economic downturns should take into account the highlighted unobserved heterogeneity.

## Appendix: Data source and variable definitions

This paper uses data from SHARE Waves 1, 2, 4, and 5 (DOIs: 10.6103/SHARE.w1.700, 10.6103/SHARE.w2.700, 10.6103/SHARE.w4.700, 10.6103/SHARE.w5.700). The SHARE data collection has been funded by the European Commission through FP5 (QLK6-CT-2001-00360), FP6 (SHARE-I3: RII-CT-2006-062193, COMPARE: CIT5-CT-2005-028857, SHARELIFE: CIT4-CT-2006-028812), FP7 (SHARE-PREP: GA N${{}^\circ }$211909, SHARE-LEAP: GA N${{}^\circ }$227822, SHARE M4: GA N${{}^\circ }$261982) and Horizon 2020 (SHARE-DEV3: GA N${{}^\circ }$676536, SERISS: GA N${{}^\circ }$654221) and by DG Employment, Social Affairs & Inclusion. Additional funding from the German Ministry of Education and Research, the Max Planck Society for the Advancement of Science, the U.S. National Institute on Aging (U01_AG09740-13S2, P01_AG005842, P01_AG08291, P30_AG12815, R21_AG025169, Y1-AG-4553-01, IAG_BSR06-11, OGHA_04-064, HHSN271201300071C) and from various national funding sources is gratefully acknowledged (see www.share-project.org).

We consider questions *sp002: ‘In the last twelve months has any family member from outside the household, any friend or neighbour given you or your partner personal care or practical household help?’* for informal care outside the household and *sp020: ‘Is there someone living in this household who has helped you regularly during the last twelve months with personal care?’* for informal care inside the household to define informal care receipt variable. A problem is that these questions do not always identify who is the person within a couple receiving care. To assign who is receiving care when there is a couple, we follow the following algorithm. Suppose care was received and there is a person in a couple with activity or mobility limitations. In that case, we assign care to this person. When both individuals have limitations, we assign care to both of them. When care was received, one respondent lives in a nursing home, and the spouse does not have health limitations, the care receipt is assigned to a nursing home resident. This leaves less than 3% observations unassigned. In these less than 3% observations, care was assigned to both members of the couple. The results presented in the paper follow this algorithm.

However, for the less than 3% unassigned observations, we tried the following alternative criteria and re-estimate our model with these alternative constructions of the dependent variable:

(i) assign care only to the person responding to the question;
(ii) assign care only to the oldest person in the couple;
(iii) assign care using additional information about health; specifically assign care to a respondent with long-term illness, and in the remaining unassigned cases, assign care to both members of the couple;
(iv) assign care using additional information about health; specifically assign care to a respondent with long-term illness, and in the remaining unassigned cases, assign care to the person responding to the question.

These four alternative criteria produced the same results as those reported in the paper based on the algorithm described in the previous paragraph.

We use the generated educational module, gv_isced, in particular, a generated *isced1997_r *variable to define our educational variable. Its potential answers are: Refusal (0.05%); Don’t know (0.01%); None (4.16%); ISCED-97 code 1 (23.78%); ISCED-97 code 2 (18.13%); ISCED-97 code 3 (29.59%); ISCED-97 code 4 (1.91%); ISCED-97 code 5 (19.78%); ISCED-97 code 6 (0.77%); Still in school (0.04%); Other (0.59%); missing value (1.18%). We discarded observations whose answer was Refusal, Don’t know, Still in school, Other and missing value. The other answers were used to built the ‘secondary education’ and ‘tertiary education’ indicator variables, leaving less than secondary as the reference category.

## Footnotes

1 See Appendix for the full citation of the data source.

2 (Costa-Font et al. 2016) also use the SHARE data. To the extent possible, we have followed the same criteria described in their paper to select the sample and built the variables we use for estimation. The sample is close to Costa-Font et al. (2016), but it does not fully coincide. In any case, our goal is not to replicate their paper but to contribute to characterize the cyclicality of informal care in the population of those countries.

3 One of the downsides of using a linear model with binary dependent variables is that, when having fixed effects, it is not approximating the Average Marginal Effect for the entire population, but for a subpopulation defined based on the temporal variation of the variables, as has been showed in Theorem 1 of Chernozhukov et al. (2013) and in Appendix of Davezies et al. (2021).

4 The BIC criteria for the number of types prefers the specification with two types over three. See  “Further discussion about heterogeneity” section for further discussion. Additionally, see Browning and Carro (2013, 2014) for some related results on the maximum number of types that can be point identified in binary choice models.

5 This relation between living with a partner and receiving care has been also found, for example, in Bonsang (2009), Gannon and Davin (2010).

6 The proportion of this group is equal to $\Pr \left( \text {type}=1|age\ge 70\right) *\Pr \left( age\ge 70\right)$ $=0.3011*0.3127$ $=0.0942$.

7 The proportion of this group is equal to $\Pr \left( \text {type}=2|age<70\right) *\Pr \left( age<70\right)$ $=0.8359*0.6873$ $=0.5745$.
